# Punch biopsies shorten time to clearance of high-risk human papillomavirus infections of the uterine cervix

**DOI:** 10.1186/s12885-018-4225-9

**Published:** 2018-03-23

**Authors:** K. U. Petry, J. Horn, A. Luyten, R. T. Mikolajczyk

**Affiliations:** 1 Department of Obstetrics and Gynecology, Klinikum Wolfsburg, Wolfsburg, Germany; 2 Institute for Medical Epidemiology, Biometrics, and Informatics, Martin-Luther-University Halle-Wittenberg, Halle, Germany; 3 Hannover Medical School, Hannover, Germany; 4 German Centre for Infection Research/ Site Braunschweig-Hannover, Braunschweig, Germany

**Keywords:** HPV infection, Clearance rate, Progression, Biopsy

## Abstract

**Background:**

The primary objective was to determine human papilloma virus (HPV) clearance rate after cervical biopsy among women with persistent high-risk HPV infection compared with spontaneous HPV clearance rate in the absence of biopsy.

**Methods:**

We collected data from a dedicated screening program of women aged 30–70 years old. Inclusion criteria for the baseline non-interventional cohort were a positive HPV test (hybrid capture 2, HC2) and normal cytology. In the baseline cohort women were followed with approximately yearly HPV-tests and cytology until HPV regressed (one negative HPV test) or interventions in the form of diagnostic biopsies or therapy. Women who had a diagnostic biopsy were included in the biopsy cohort and followed until HPV regression or therapy. Observed HPV regression rates and time to HPV regression were compared between baseline and biopsy cohorts. For the comparison, we used Fisher’s exact test for the HPV regression rates and interval-censored, accelerated failure time model for time to HPV regression.

**Results:**

Among the 1079 women included in the baseline cohort, 499 (46.3%) had HPV clearance and 475 were referred for colposcopy with biopsy. The biopsy cohort comprised all women who were not treated and had at least one HC2 test after biopsy (201/475; 42.3%). Of those, 138 (68.7%) experienced HPV regression. In the biopsy cohort, time to clearance of HPV infection was approximately halved (0.46, 95% CI 0.38–0.56) compared with the baseline cohort. This result was robust in a wide range of sensitivity analyses.

**Conclusions:**

A higher proportion of women cleared their HPV infection, and time to HPV clearance was shorter in the biopsy cohort than in the baseline cohort. It is reassuring for clinicians to know that conservative management of patients with HPV persistency is successful when colposcopy with biopsies excludes high-grade disease.

## Background

It is well established that infection of the uterine cervix with high-risk human papillomavirus (HPV) types needs to persist for many years or even decades before invasive cervical cancer develops. There are different approaches to estimate the risk of malignant progression associated with HPV infections and the overall risk of cervical cancer. The life-time risk for cervical cancer reaches 6.5% in some areas without cervical cancer screening [1]. However, in most industrial countries with cervical cancer screening programs the risk of developing cervical cancer is usually calculated step by step according to the widely accepted model of carcinogenesis from persistent HPV infection to precursor lesions, classified as cervical intraepithelial neoplasia (CIN), and finally cancer. CIN3 is an endpoint in many trials and is the confirmed true precursor of cervical cancer [2].

Studies on the clinical course of HPV infections of the uterine cervix require colposcopy with histological assessment (biopsy) at recruitment because cytology or colposcopic impression alone may under- or overestimate the grade of associated CIN lesions [3]. Although taking biopsies may alter the clinical course of cervical lesions [4], the rate of spontaneous regression of CIN3 lesions is controversial, and there is no evidence to support theories that procedure-related immune activation can lead to regression [5].

Long-term observational studies of women with incident or prevalent HPV infections suggest a typical clearance pattern with a high regression rate in the first year of observation and lower regression rates in subsequent follow-up years [6]. It was our a priori hypothesis that taking biopsies late during follow-up would change this pattern resulting in increased rather than decreased regression rates. Such a therapeutic effect of the biopsy procedure would complement the effectiveness of observational management of women with HPV persistency when colposcopy has been used to exclude high-grade cervical lesions. The primary objective of this study was to determine HPV clearance rate after cervical biopsy among women with persistent high-risk HPV infection compared with the spontaneous HPV clearance rate without biopsy.

## Methods

The Wolfsburg pilot project for better prevention of cervical cancer with Primary HPV Screening (WOLPHSCREEN) has been described in detail elsewhere [7]. In brief, non-hysterectomized women aged 30 to 70 years covered by ‘Deutsche BKK’ or ‘Audi BKK’ health insurance, are screened using Pap smear and HPV (Hybrid Capture 2, HC2) tests. Screening is repeated after 5 years for participants with normal Pap smears and negative HC2 tests, while those with abnormal Pap smears and positive HC2 test results are immediately referred for colposcopy. Cervical swabs using a brush are taken from all participants. HPV testing is done routinely with HC2 according to the standard protocol recommended by the manufacturer (Qiagen, Hilden, Germany). HC2 detects high-risk HPV types 16, 18, 31, 33, 35, 39, 45, 51, 52, 56, 58, 59, and 68. The threshold for a positive HC2 result is 1 RLU (relative light unit). Cervical smears are classified in accordance with the Munich Cytological Classification [8]. Abnormal smear test results were defined as Pap IIw or IIp, which are equivalent to, or worse than, atypical cells of undetermined significance (ASC-US) in the Bethesda system.

Women with positive HC2 tests and normal cytology are followed in yearly intervals. After each year, HC2 testing is repeated and those women with a persistent positive HC2 test may be referred for colposcopy. The colposcopy protocol mandates biopsies for all women with a persistent, positive HC2 test result after 1 year, including those with normal Pap smear test results; therefore, more than 90% of referred women undergo histological assessment.

The ethics committee of Lower Saxony in Hannover was consulted but approval and/or trial registration was not required because statutory health insurances Deutsche BKK and Audi BKK use the pilot project as their routine screening program. WOLPHSCREEN is managed by Klinikum Wolfsburg, the central database is located in the department of gynecology. The contract between health insurances, gynecologists in private practice, other partners, and Klinikum Wolfsburg encourages the use of WOLPHSCREEN data for research and gives all rights for analyses and publications to the head of the department of obstetrics and gynecology as leader of the scientific team. Participants need to give written consent to be included in WOLPHSCREEN. The written consent includes an agreement that pseudonymized data and left-over material may be used for research.

We followed women aged 30 to 70 years with a positive HC2 test and normal Pap smear at recruitment and at least one further HC2 prior to any intervention or colposcopy to determine the natural course of HPV infection. Women stay in the baseline cohort until regression of HPV infection (the first negative HC2 result), cervical biopsy or therapy, or their last HC2 test, whichever occurs first. Women from the baseline group with a positive HC2 test for at least 1 year can be subsequently referred for colposcopy/biopsy and then included in the ‘biopsy cohort’. In the biopsy cohort, women were observed until HPV regression, further biopsy or therapy, or their last HC2 test (the same criteria as in the baseline cohort). The ‘biopsy cohort’, therefore, comprises a subsample of women from the baseline group at a different time point.

### Statistical analysis

First, the distribution of sociodemographic and clinical characteristics was compared for both cohorts by means of tabulation and chi-squared test. In this analysis, we did not exclude women who contributed more than one baseline period (second round of testing followed 5 years after the first and each of the testing rounds initiated its own follow-up of patients; thus, women who cleared HPV infection in the first follow-up round could re-enter the baseline cohort when they fulfilled the inclusion criteria in the second round of screening). Furthermore, we ignored that women included in the biopsy cohort were a subsample of women in the baseline cohort. In the next step, we used an accelerated failure time (AFT) regression model to compare regression of HPV infection in the biopsy cohort with the baseline cohort. AFT is an alternative approach to Cox regression [9]. In the interpretation of the model estimates the parameters apply directly to time and give information if the process is faster or slower than in the comparison group. The AFT models are less commonly used than Cox models, but for the current analysis had the advantage of being able to handle interval-censored data [10]. The exact time of HPV regression is not known: it had to occur somewhere between the last positive and the first negative HC2 test, generating interval-censored data. We assumed that the time to HPV regression is distributed exponentially, i.e. there is a constant regression rate during the follow up. We also tested other distributions including Weibull or normal, but the exponential distribution displayed the best fit and was the most stable (after adding a small correction of 0.01 to avoid problems with estimates equal to 0). While previous observations suggested that regression is slower when HPV infection persists for longer, the only consequence of assuming an equal regression rate during follow up is that positive biopsy effects are estimated conservatively. The results were also visualized as Kaplan-Meier curves for clearance of HPV infection: for this analysis in a simplified fashion, we assumed that regression occurred at the time point of the first negative test, which causes conservative bias.

The effects of changing the inclusion criteria for baseline and/or biopsy cohort were studied in four sensitivity analyses (1) further biopsies were no longer considered a censoring event in the biopsy cohort and were included in the analysis; (2) women with ASC-US or more at the time of inclusion in the biopsy cohort were excluded after biopsy; (3) women developing CIN3 at any point during follow-up were excluded in both cohorts; and (4) only the first follow-up period per woman was included in the analysis.

## Results

**Fig. 1:**
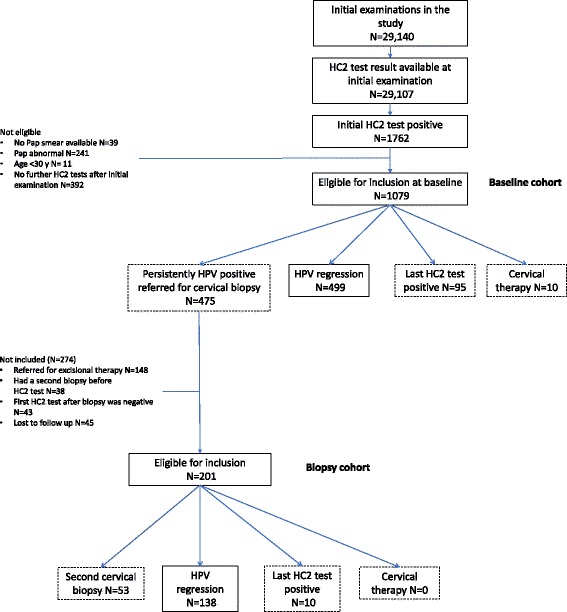
Flow chart of the population of women included in the study. Women from the baseline cohort move to the biopsy cohort when they are referred for colposcopy

From 29,140 follow-up periods available for 22,029 women, 1079 follow-up periods matched all inclusion criteria for the baseline cohort (HPV test positive, normal Pap smear at entry, and age 30–70 years; see Fig. 1). During follow-up, 475 women (44.0%) were referred for colposcopy and biopsy because of persistent HC2 positive test for at least 1 year. Women who contributed to the biopsy cohort were slightly younger than all women in the baseline cohort (Table 1). Because women with abnormal Pap smears were excluded from baseline cohort, abnormal smears were observed only in the biopsy cohort.

**Table 1:** Description of the participants contributing to the baseline and biopsy cohorts. (both cohorts consist of HPV positive women

	Baseline cohort (*n* = 1079 women)	Biopsy cohort (*n* = 201 women) *
Age at cohort entry		
30–39	539 (50.0%)	119 (59.2%)
40–49	303 (28.1%)	47 (23.4%)
50–59	134 (12.4%)	23 (11.4%)
60–69	64 (5.9%)	7 (3.5%)
70+	39 (3.6%)	5 (2.5%)
Smoking (at initial examination)		
Never	551 (51.1%)	98 (48.8%)
Current	384 (35.6%)	82 (40.8%)
Ex	110 (10.2%)	21 (10.4%)
Unknown	34 (3.1%)	0 (0.0%)
Pap smear at cohort entry		
Normal	1079 (100%)	142 (70.7%)
ASC-US	0	23 (11.4%)
ASC-H	0	3 (1.5%)
LSIL	0	33 (16.4%)

*All women in the biopsy cohorts are also included in the baseline cohort

In the baseline cohort, 499 women (46.3%) showed spontaneous HPV regression without any intervention, 10 (0.9%) underwent immediate excisional therapy, and 95 (8.8%) had persistent HPV infection at the end of follow up, but did not have immediate colposcopy for a variety of reasons (non-compliance, yet to accept referral to colposcopy, became HPV negative or had colposcopy done after the analysis period after the end of this analysis, or lost to follow up). The remaining 475 women were referred for biopsy. Among them 201 (42.3%) had follow-up periods with additional HC2 test results and were included in the ‘biopsy cohort’. Of these, 138 (68.7%) experienced HPV clearance and 53 (26.4%) required further biopsies (see Fig. 1).

Among the 274 women who underwent colposcopy with biopsy, but were not included in the biopsy cohort: a) 148 women were referred for excisional treatment without further HPV tests between biopsy and treatment; b) 38 women had a second biopsy before subsequent HPV testing (in these women HPV infection cleared in 27 cases); these 38 cases were included in the sensitivity analysis ‘multiple biopsies allowed’; c) 43 cases whose last HPV test before transition to the biopsy cohort was positive and their first HPV test after the biopsy was negative; these cases represent spontaneous regression, however, we adopted this conservative approach (reducing the possible effects of biopsies) because HPV testing was not done at the time of biopsy and, therefore, it was not clear if clearance occurred in the baseline cohort or after the transition to the biopsy cohort; d) the remaining 45 women were lost to follow-up, mostly because the time for their next scheduled visit was not reached yet at the time of our analysis.

**Fig. 2:**
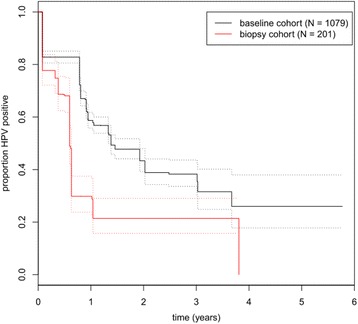
Kaplan Meier plot of clearance of HPV infection in comparison between baseline and biopsy cohort

The proportion of women who cleared HPV infection was consistently higher in the biopsy cohort than in the baseline cohort (Fig. 2). In the multivariable analysis, the time to regression in the biopsy cohort was less than halved when compared with the baseline cohort (0.46, 95% confidence interval 0.38–0.56) (Table 2, first column). Smoking was associated with an approximately 50% (46% current; 53% past) longer time to regression, while regression was faster in older women, corresponding to a 6% decrease per 5 years of life. The results were similar in the various sensitivity analyses (Table 2, columns 2–5), most notably even exclusion of CIN3 cases in both biopsy and baseline cohorts did not change this result.

**Table 2:** Variables associated with time to HPV regression (multivariable interval censored accelerated failure time regression model with exponentially distributed regression times, estimates how many times shorter or longer is the time to regression in comparison to the reference category; mutually adjusted for all variables in the table)

	Main analysis	Multiple biopsies allowed	Max. Pap normal after biopsy	All persons with CIN3+ removed	Only first follow-up included
	Estimate (95% CI)	Estimate (95% CI)	Estimate (95% CI)	Estimate (95% CI)	Estimate (95% CI)
Biopsy cohort vs. baseline cohort	0.46 [0.38;0.56]	0.61 [0.51;0.73]	0.41 [0.33;0.52]	0.48 [0.40;0.59]	0.45 [0.36;0.55]
Age (per 5 years)	0.94 [0.91;0.98]	0.94 [0.91;0.97]	0.95 [0.91;0.98]	0.96 [0.92;0.99]	0.95 [0.93;0.98]
Smoking (ref = Never)					
Current	1.46 [1.22;1.74]	1.47 [1.24;1.74]	1.48 [1.23;1.78]	1.44 [1.20;1.72]	1.42 [1.22;1.78]
Past	1.53 [1.14;2.05]	1.55 [1.17;2.06]	1.43 [1.06;1.93]	1.46 [1.09;1.96]	1.47 [1.04;1.94]
Unknown	0.65 [0.42;1.02]	0.77 [0.50;1.18]	0.65 [0.41;1.02]	0.69 [0.44;1.07]	0.86 [0.40;1.85]

*CI* confidence interval

## Discussion

Overall, the results confirm our a priori hypothesis that taking biopsies increases the clearance rate of high-risk HPV infections. The biopsy cohort comprised women with persistent HPV infection from the start of observation for at least 1 year; despite this persistent infection, we observed a faster regression rate of HPV infections in the biopsy cohort than among the baseline cohort. Our data indicate a direct impact of taking biopsies on the subsequent clinical course of HPV infection. In studies examining the clinical course of prevalent HPV infections, the majority of those reporting high regression rates during the first year of observation referred all or at least a substantial proportion of participants for colposcopy with biopsy at the time of recruitment or shortly thereafter [6, 11–13]. This likely biased the estimates towards higher regression rates compared with no intervention.

The strengths of this study are the large sample size and its population-based character. Despite the large size, all investigations were conducted by a single team using a standardized protocol. The main limitation is that the data do not come from a clinical trial but from real-life cervical cancer screening. Therefore, the probability of censoring (due to treatment or biopsy) is not independent of the results of the HPV tests. In many cases, it is not clear if this under- or overestimates the effect of biopsy, but in cases where the direction of effect is clear, it results in an underestimation and, therefore, we consider our final estimate of biopsy effects as conservative. Furthermore, women screened in the WOLPHSCREEN program are followed up consistently and systematically according to published protocols [7]. Given the limited sample size, we were not able to investigate whether women with abnormal cytology and HPV persistence experienced different effects of biopsy than women with normal cytology and HPV persistence. Importantly, HPV clearance and subsequent reinfection between two HPV tests cannot be differentiated from HPV persistence.

HPV persistency over one or more years identifies women at increased risk for long-term viral persistence and the development of CIN3+ [13, 14]. By definition, the biopsy cohort comprised women with HPV persistence for at least 1 year and a higher frequency of risk factors for HPV persistence than in women in the baseline cohort. The proportion of abnormal Pap smear results at the time of biopsy was higher than seen at baseline. Despite these differences, the regression was faster in the biopsy cohort than in the baseline cohort. Two other known factors associated with differences in regression rates were considered in the analysis: the biopsy cohort included women who were younger on average and more smokers.

The findings were also consistent in the sensitivity analyses. The inclusion of follow-up periods with multiple biopsies, most likely indicating HPV persistence, introduces potential bias because these cases were included only in the biopsy cohort and not in the baseline cohort. Consequently, and as expected, in the sensitivity analyses allowing multiple biopsies, the estimates for the effect of biopsy were smaller (indicative of a bias in a conservative direction), but still significant. The advantage of HPV screening is the detection of CIN3 lesions and cancers (CIN3+) that are missed by cytology. These cases of underlying CIN3+ in women with HPV persistence are assumed to have a very low regression rate. As the risk of CIN3+ was higher in the baseline cohort, we did a sensitivity analysis that excluded all cases of CIN3+ in all cohorts to avoid a possible bias related to the different nature of these cases. However, we still observed an increased regression rate of formerly persisting HPV infections in the biopsy cohort compared with the prevalent HPV infections in the baseline cohort. The finding of faster clearance of persistent HPV infection after biopsy among older women is unexpected and intriguing. Previously, we have shown that HC2-testing with a sensitive relative light unit threshold (RLU 1.0) seems reliable even in older women because of cross-hybridization with some potentially oncogenic HPV subtypes [15]. Nevertheless, we do not have a good explanation why the observed clearance is even better in older women.

Our findings are relevant for clinicians and patients because they suggest that observational management of women with HPV persistency is an effective approach. When high-grade disease is excluded by colposcopy with biopsies, the remaining risk of CIN3 during long-term observation is very low [7, 16], and, as shown here, HPV clearance can be expected in the majority of cases during short- to medium-term follow-up. Using additional biomarkers in the screening triage may improve cervical cancer detection and potentially reduce total costs [17].

## Conclusions

We conclude that all studies of the natural history of HPV face a dilemma. On the one hand, the exact identification of HPV-associated disease depends on high-quality diagnoses during follow-up, which in the case of cervical disease are based on colposcopy; however, the accuracy of colposcopy is determined by an appropriate number of punch biopsies [16, 18]. On the other hand, colposcopy with biopsy appears to have a therapeutic effect on CIN [4] and, as shown here, on HPV persistency. This presents researchers with a conundrum of either measuring the clinical course of HPV infections that is not truly natural, but based on best diagnoses, or to define the clinical course without relying on colposcopy with histological assessment.

## Abbreviations

AFT: Accelerated failure time
ASC-US: Atypical cells of undetermined significance
CIN: Cervical intraepithelial neoplasia
HC2: Hybrid capture 2
HPV: Human papilloma virus
WOLPHSCREEN: Wolfsburg pilot project for better prevention of cervical cancer with Primary HPV Screening
